# Beneficial effects of physical activity in an HIV-infected woman with lipodystrophy: a case report

**DOI:** 10.1186/1752-1947-5-430

**Published:** 2011-09-05

**Authors:** Edmar Lacerda Mendes, Alynne Christian Ribeiro Andaki, Ciro José Brito, Cláudio Córdova, Antônio José Natali, Paulo Roberto dos Santos Amorim, Leandro Licursi de Oliveira, Sérgio Oliveira de Paula, Eugene Mutimura

**Affiliations:** 1 Programa de Pós-Graduação em Biologia Celular e Estrutural, Laboratório de Imunovirologia Molecular, Universidade Federal de Viçosa/MG, Brasil; 2 Mestrado em Educação Física, Departamento de Ciências do Esporte, Neafisa, Universidade Federal do Triângulo Mineiro/MG, Brasil; 3 Programa de Pós-Graduação em Ciência da Nutrição, Universidade Federal de Viçosa/MG, Brasil; 4 Laboratório de Estudos em Educação Física e Saúde, Universidade Católica de Brasília/DF, Brasil; 5 Departamento de Educação Física, Universidade Federal de Viçosa/MG, Brasil; 6 Laboratório de Imunovirologia Molecular, Universidade Federal de Viçosa/MG, Brasil; 7 Women's Equity in Access to Care and Treatment and Kigali Health Institute, Kigali, Rwanda

## Abstract

**Introduction:**

Lipodystrophy is common in patients infected with human immunodeficiency virus receiving highly active antiretroviral therapy, and presents with morphologic changes and metabolic alterations that are associated with depressive behavior and reduced quality of life. We examined the effects of exercise training on morphological changes, lipid profile and quality of life in a woman with human immunodeficiency virus presenting with lipodystrophy.

**Case presentation:**

A 31-year-old Latin-American Caucasian woman infected with human immunodeficiency virus participated in a 12-week progressive resistance exercise training program with an aerobic component. Her weight, height, skinfold thickness, body circumferences, femur and humerus diameter, blood lipid profile, maximal oxygen uptake volume, exercise duration, strength and quality of life were assessed pre-exercise and post-exercise training. After 12 weeks, she exhibited reductions in her total subcutaneous fat (18.5%), central subcutaneous fat (21.0%), peripheral subcutaneous fat (10.7%), waist circumference (WC) (4.5%), triglycerides (9.9%), total cholesterol (12.0%) and low-density lipoprotein cholesterol (8.6%). She had increased body mass (4.6%), body mass index (4.37%), humerus and femur diameter (3.0% and 2.3%, respectively), high-density lipoprotein cholesterol (16.7%), maximal oxygen uptake volume (33.3%), exercise duration (37.5%) and strength (65.5%). Quality of life measures improved mainly for psychological and physical measures, independence and social relationships.

**Conclusions:**

These findings suggest that supervised progressive resistance exercise training is a safe and effective treatment for evolving morphologic and metabolic disorders in adults infected with HIV receiving highly active antiretroviral therapy, and improves their quality of life.

## Introduction

The use of highly active antiretroviral therapy (HAART) reduces morbidity and mortality rates and improves the wellbeing of patients who are human immunodeficiency virus (HIV) seropositive [1]. However, the use of HAART is associated with changes in body fat deposits and metabolic alterations. The term 'lipodystrophy' is traditionally used to describe various morphological changes related to fat redistribution, for example, lipoatrophy (the loss of fat) and lipohypertrophy (fat accumulation). Lipoatrophy and lipohypertrophy may occur separately or in combination in one individual [2]. In some patients infected with HIV, the changes are characterized by increased central body fat accumulation, including visceral adipose tissue. This can present as abdominal obesity or, more rarely, an accumulation of fat in the dorsocervical region, called 'buffalo hump'. Disturbances in body fat distribution may be accompanied by metabolic disorders including glucose intolerance, insulin resistance, hypertension and dyslipidemia [3]. Morphologic and metabolic disturbances result in impaired body image and a risk of cardiovascular diseases and diabetes.

Disturbances in body fat distribution may also be accompanied by lipoatrophy, which typically involves loss of subcutaneous fat from the face, arms, legs and buttocks. Although the combination of visceral adiposity and metabolic disorders is not unique to the HIV population receiving HAART, its pathogenesis and clinical presentation in these patients seem to be different from that of the general population [4]. In individuals who are HIV seronegative, regular physical activity is associated with favorable changes in blood lipids, particularly an increase in plasma high density lipoprotein (HDL) cholesterol, a reduction in plasma triglycerides and the ratio between total cholesterol and HDL cholesterol [5]. Based on this premise, the current guidelines recommend physical activity as non-pharmacological treatment for individuals who are HIV-positive with dyslipidemia who are receiving HAART [6], and exercise training reportedly minimizes depressive symptoms in women infected with HIV [7]. There are few studies examining the effects of aerobic and resistance exercise training on lipodystrophy and quality of life in adults infected with HIV, suggesting the need to test further these interventions in people of all races who are HIV-positive.

## Case presentation

A 31-year-old Latin-American Caucasian woman infected with HIV through a heterosexual relationship with a partner received treatment at the Health Promotion Centre (HPC) of Conselheiro Lafaiete, Brazil. She had oral candidiasis and had started to develop depression. These were controlled with the use of ketoconazole 400 mg and fluoxetine 20 mg daily, respectively. She was vaccinated against hepatitis B and started combined therapy with lamivudine (3TC) 150 mg and zidovudine (AZT) 300 mg plus nevirapine 200 mg twice daily when her viral load was over 500,000 copies, her CD4+ level was 33 cells/μL and CD8+ level was 287 cells/μL. This regimen improved our patient's health. Seven months later, there was a reduction in viral load to 1084 copies, an increase of CD4+ by 130 cells/μL and CD8+ by 503 cells/μL. 3TC/AZT/NPV were started but replaced for 3TC/d4T/NPV due to anemia. With the new regimen, the viral load became undetectable, with an increase of CD4+ to 212 cells/μL and CD8+ to 762 cells/μL. She continued the recommended regular medical visits (Table 1).

**Table 1:** History of medical visits performed by the HPC.

Medical visits	Therapeutic regimen	Viral load	CD4+	CD8+	Presence of lipodystrophy
1	AZT/3TC/NVP	>500,000	33	297	No
2	d4T/3TC/NVP	1084	130	503	No
3	d4T/3TC/NVP	<50	212	762	No
4	d4T/3TC/NVP	<50	319	1063	No
5	d4T/3TC/NVP	<50	477	1253	No
6	d4T/3TC/NVP	<50	663	1245	Yes
7	d4T/3TC/NVP	<50	547	1322	Yes
8	d4T/3TC/NVP	<50	675	1130	Yes

During the sixth medical visit, our patient reported concerns about a loss of muscle mass. She was clinically diagnosed as having lipoatrophy of the upper and lower limbs. She showed increased dissatisfaction with her appearance during her next medical visit.

After another 10 months, our patient signed an informed consent form to voluntarily participate in this study after guided ethical information was provided. This study was approved by the Ethics Committee in Human Research of the Federal University of Viçosa-Minas Gerais. All measurements were performed after a 24-hour abstention from strenuous exercise, and blood samples were collected after a 12-hour fast. At baseline and at 12 weeks, anthropometric measures, maximum oxygen uptake (V_O2max_), blood samples and quality of life measures were assessed. Strength tests were performed at baseline, six and 12 weeks.

The exercise program consisted of 12 weeks of supervised exercise (preceded by two weeks of adaptation) performed three times per week on non-consecutive days (Table 2). During the period of adaptation, aerobic training was performed on a treadmill with crescent intensity, ranging from 50% to 60% of the heart rate reserve (HRR), as determined by the maximal treadmill exercise test. Resistance training, three sets of six to ten repetitions, was performed at 60% of one maximum voluntary contraction (1-RM). All training sessions were performed at the Centre for Research (accredited by the HPC), supervised by one of the authors of this study (ELM). The cardiovascular exercise was performed on a treadmill with crescent intensity, ranging from 50% to 80% of HRR. We used the American College of Sports Medicine (ACSM) metabolic equation for measurement of cardiorespiratory fitness, using the "walking" equation, to estimate V_O2max _for the modified Bruce protocol: V_O2max_(mL/kg/min) = (speed(m/min) × 0.1+((grade(decimal) × speed(m/min) × 1.8)+3.5. Heart rate was monitored during all sessions to ensure that proper training intensity was maintained. Three sets of eight to ten repetitions were performed at 80% of 1-RM according to the guidelines of the ACSM [8]. Six resistance training exercises, targeting the large muscle groups of the body, were performed in the following order: seated leg press, chest press, leg curl, pulldown, leg extension, seated rows.

**Table 2:** Exercise intervention design

Weeks	Sessions/week	Session	Aerobic	Resistance training
0	3	1	20 minutes 60% to 70% HRR	6 to 8 reps 70% 1-RM
		2	20 minutes 60% to 70% HRR	8 to 10 reps 70% 1-RM
		3	20 minutes 60% to 70% HRR	8 to 10 reps 70% 1-RM
1 to 12	3	1	20 minutes 70% to 80% HRR	8 to 10 reps 80% 1-RM
		2	20 minutes 70% to 80% HRR	8 to 10 reps 80% 1-RM
		3	20 minutes 70% to 80% HRR	8 to 10 reps 80% 1-RM

reps: repetitions

Our patient's anthropometric measures, body composition, biochemical and immune characteristics are shown in Table 3. For body fat assessment, we used the methodology proposed by Florindo *et al*. [9]. Positive changes were observed in her body composition at the end of the intervention. The central subcutaneous fat and peripheral subcutaneous fat showed a reduction of 18.5 mm and 3 mm, respectively. This result equated to a reduction of 22.8% in total subcutaneous fat. Body density was calculated using the equation of Jackson *et al*. [11] for women, and this result was then used to calculate body fat percentage, using the equation of Siri [10]. Our patient's body fat percentage decreased 18.5% due to the loss of 2.1 kg of body fat. Her fat-free mass (FFM) increased by 4.9 kg. Both her femur and humerus diameters, measured using a digital paquimeter, increased by 2 mm. Blood samples were measured in a clinical laboratory. There was a reduction in triglycerides (9.9%), total cholesterol (12%), and low-density lipoprotein cholesterol (LDL) (8.6%) and an increase in HDL cholesterol (16.7%). Lymphocytes and neutrophils increased by 97 and 55 cells/mm^3^, respectively. These results were associated with an increase of 2.9% in total leukocytes.

**Table 3:** Values determined at baseline and after 12 weeks of exercise intervention

Variable	Pre-test	Post-test	Change	Percentage change
Body mass, kg	61.2	64	2.8	4.6
Body mass index, kg/m^2^	22.21	23.23	1.02	4.6

Circumferences, cm				

Neck circumference	35	33.7	-1.3	-3.7
Chest circumference	93	91.5	-1.5	-1.6
Waist circumference	82	78.3	-3.7	-4.5
Mid-arm circumference	27	29.4	2.4	8.9
Forearm circumference	23.5	24.4	0.9	3.8
Thigh circumference	50	51.2	1.2	2.4
Calf circumference	33.5	34.2	0.7	2.1
Waist-to-hip ratio (WHR)	0.91	0.87	-0.04	-4.3

Body composition				

TSF, mm	116.0	94.5	-21.5	-18.5
CSF, mm	88	69.5	-18.5	-21.0
PSF, mm	28	25	-3	-10.7
Fat, %	24.1	19.7	-4.4	-18.3
Body fat, kg	14.7	12.6	-2.1	-12.5
FFM, kg	46.5	51.4	4.9	12.7
Femur, cm	8.4	8.6	0.2	2.4
Humerus, cm	6.4	6.6	0.2	3.1

Metabolic and immunological values				

Triglycerides, mg/dL	142	128	-14	-9.9
Total cholesterol, mg/dL	225	198	-27	-12.0
LDL cholesterol, mg/dL	162	148	-14	-8.6
HDL cholesterol, mg/dL	36	42	6	16.7
HbA_1c_	6.12	6.06	-0.06	-1.0
Fasting glucose, mg/dL	88	90	2	2.3
Leukocytes, cells/m^3^	6900	7100	200	2.9
Lymphocytes, cells/m^3^	2463	2560	97	3.9
Neutrophils, cells/m^3^	3760	3815	55	1.5
Basophils, cells/m^3^	82.8	81.4	-1.4	-1.7
Monocytes, cells/m^3^	448.5	455	6.5	1.4
Platelets, cells/m^3^	279,000	278,600	-400	-0.1

CSF: subcutaneous fat; HbA_1c_: glycated fasting hemoglobin; PSF: peripheral subcutaneous fat; TSF: total subcutaneous fat

Fitness characteristics indicate that her V_O2max _and exercise duration increased by 8mL/kg/minute and three minutes, respectively (Table 4). Her maximum dynamic muscle strength increased for all six trained muscle groups (range of increase 54.5% to 83.3%) (Table 4).

**Table 4:** Fitness and strength measures

Variables	Pre-test	Post-test	Change	Percentage change
Fitness measures:				

V_O2max_, mL/kg/min	24	33	8	33.3
exercise duration, min	8	11	3	37.5

Strength measures, kg				

leg curl	8	14	6.0	75.0
pulldown	16	28	12.0	75.0
seated leg press	30	55	25.0	83.3
chest press	12	20	8.0	66.7
seated rows	22	34	12.0	54.5
leg extension	18	25	7.0	38.9

**Figure 1:**
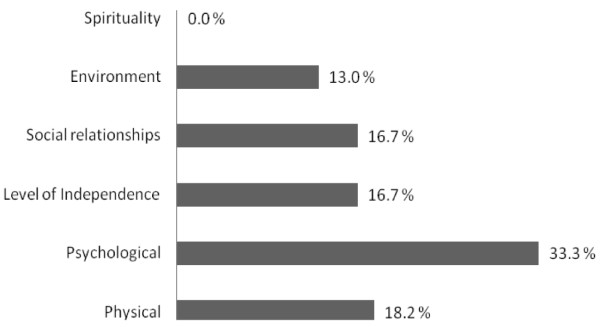
**Percentage change in quality of life domains**.

Quality of life measures were assessed using the World Health Organization Quality of Life assessment instrument in patients with HIV best available reference techniques. These measures improved mainly for the psychological domain, followed by the physical domain, her level of independence, personal relationships and environment (Figure 1).

## Discussion

We have shown that a 12-week exercise program results in weight gain and improved body composition changes in a woman infected with HIV with lipodystrophy syndrome. Body composition, lipid profile, maximum oxygen consumption, strength, and quality of life improved as observed in previous studies [2,12]. Changes in body composition were also similar to those seen in the case study of a man infected with HIV reported by Roubenoff *et al*. [13]. Several studies involving exercise training for adults who are HIV-positive have also reported a reduction in body fat composition and increased FFM [2,7,12].

The reduction in the measurements of her chest and waist, and increased diameter of her mid-arm, forearm, thigh and calf could have been crucial to changing our patient's perception of her body image. Quality of life measures in the physical and psychological domains improved after exercise training, as reported by others [14].

There were decreases in visceral and subcutaneous fat in the central region of her body, which has been associated with insulin resistance, dyslipidemia, hypercholesterolemia and risk for cardiovascular disease. Although we have not used methods of radiologic imaging, the intervention resulted in WC reduction above the recommended cut-off point for women (WC ≥ 80 cm) [15]. Consequently, her lipid profile changed positively after exercise training with a reduction in triglycerides, total cholesterol, LDL cholesterol and an increase in HDL cholesterol.

Carr *et al*. [16] similarly reported that low body weight before commencing HAART is associated with lactic acidosis attributed to nucleoside transcriptase reverse inhibitors; this provides a foundation for osteopenia in men who are HIV-positive. Our results reinforce the importance of exercise training on the maintenance of bone mass at baseline levels.

Our findings can only be applied to this case, but suggest that exercise training might be safe for treatment of lipodystrophy-related body changes (mainly central adiposity) and improve the lipid profile without adverse changes in immunologic outcomes. However, this is only a case study and the exact contribution of each type of exercise (resistance and aerobic training) needs to be ascertained in further studies.

Her increased oxygen consumption and strength were the most significant results. This can be explained by both the increase in lean mass, of 4.9kg, and the fact that after infection our patient was deprived of social contact and participated more in regular physical exercise. Power *et al*. [17] reported that lipodystrophy-related body changes result in physical and psychological impairment, ranging from bodily discomfort to low self-esteem and depression, and this likely influenced our patient's increased participation in exercise training to gain psychological comfort. Lipodystrophy-related morphological changes result in individuals narrowing their social world, and in some cases result in social isolation. Thus, these body changes resulting in a 'slim body' in the HIV-positive population are commonly associated with loss of lean body mass. We did not measure oxygen consumption directly, but instead used a prediction equation [18] not previously validated in a population with HIV infection.

Power *et al*. [17] reported that three years of HAART negatively affected psychosocial wellbeing, mainly due to changes in body image. Préau *et al*. [19] provided evidence that the reduced quality of life in women is associated with the accumulation of body fat. Our results support these prior findings, and further suggest that a reduction in body fat was directly related to the improvements in their quality of life.

## Conclusions

Regular exercise training improved physical fitness and was effective and safe in mitigating changes associated with lipodystrophy and dyslipidemia in a woman infected with HIV. These preliminary results suggest that supervised progressive resistance exercise is an inexpensive and practical treatment for lipodystrophy body changes and dyslipidemia, and improves quality of life in adults infected with HIV.

## Patient's perspective

I think that exercise improved my health and it is very important to maintain it. I noted that it greatly improved my body; my paunch has decreased in size but I still want more. I think the main change was the increase in my weight and my legs.

## Consent

Written informed consent was obtained from the patient for publication of this case report and any accompanying images. A copy of the written consent is available for review by the Editor-in-Chief of this journal.

## Competing interests

The authors declare that they have no competing interests.

## Authors' contributions

ELM and CJB dealt directly with our patient, ordered the laboratory examinations and decided on the exercise intervention design. ELM, ACRA, CJB, CC, LLO and SOP analyzed and discussed the data as well as prepared the manuscript. AJN, PRSA, EM and SOP reviewed clinical data and provided scientific input on writing the manuscript. All authors read and approved the final manuscript.
